# Protein Synthesis Attenuation by Phosphorylation of eIF2α Is Required for the Differentiation of *Trypanosoma cruzi* into Infective Forms

**DOI:** 10.1371/journal.pone.0027904

**Published:** 2011-11-16

**Authors:** Renata R. Tonelli, Leonardo da Silva Augusto, Beatriz A. Castilho, Sergio Schenkman

**Affiliations:** Departamento de Microbiologia, Imunologia e Parasitologia, Escola Paulista de Medicina, Universidade Federal de São Paulo, São Paulo, Brasil; French National Centre for Scientific Research, France

## Abstract

Chagas' disease is a potentially life-threatening illness caused by the unicellular protozoan parasite *Trypanosoma cruzi.* It is transmitted to humans by triatomine bugs where *T. cruzi* multiplies and differentiates in the digestive tract. The differentiation of proliferative and non-infective epimastigotes into infective metacyclic trypomastigotes (metacyclogenesis) can be correlated to nutrient exhaustion in the gut of the insect vector. *In vitro*, metacyclic-trypomastigotes can be obtained when epimastigotes are submitted to nutritional stress suggesting that metacyclogenesis is triggered by nutrient starvation. The molecular mechanism underlying such event is not understood. Here, we investigated the role of one of the key signaling responses elicited by nutritional stress in all other eukaryotes, the inhibition of translation initiation by the phosphorylation of the eukaryotic initiation factor 2α (eIF2α), during the *in vitro* differentiation of *T. cruzi*. Monospecific antibodies that recognize the phosphorylated Tc-eIF2α form were generated and used to demonstrate that parasites subjected to nutritional stress show increased levels of Tc-eIF2α phosphorylation. This was accompanied by a drastic inhibition of global translation initiation, as determined by polysomal profiles. A strain of *T. cruzi* overexpressing a mutant Tc-eIF2α, incapable of being phosphorylated, showed a block on translation initiation, indicating that such a nutritional stress in trypanosomatids induces the conserved translation inhibition response. In addition, Tc-eIF2α phosphorylation is critical for parasite differentiation since the overexpression of the mutant eIF2α in epimastigotes abolished metacyclogenesis. This work defines the role of eIF2α phosphorylation as a key step in *T. cruzi* differentiation.

## Introduction

The reversible phosphorylation of the α-subunit of the eukaryotic translation initiation factor 2 (eIF2α) is a major survival response, conserved from yeast to mammals, and plays a significant role when cells must respond to environmental stresses. The process of translation initiation in eukaryotes consists of binding of methionyl-initiator tRNA and mRNA to the 40S ribosomal subunit, pairing the Met-tRNA_i_^Met^ with the AUG start codon and joining of the 60S ribosomal subunit to form the 80S elongating ribosome. In this process, eIF2 plays an important role in delivering Met-tRNA_i_^Met^ to the translational machinery. This is achieved when the ternary complex formed by eIF2 bound to GTP and Met-tRNA_i_^Met^ is transferred to the 40S subunit. For the assembly of the 80S complex on the AUG initiator codon, eIF2 is released in the form of the binary inactive eIF2-GDP complex. For another round of translation initiation, eIF2 must be converted to its eIF2-GTP active form. This recycling event is mediated by eIF2B, a nucleotide guanine exchange factor (GEF) [1]. When eIF2α is phosphorylated specifically at Ser^51^, eIF2-GDP becomes a competitive inhibitor of eIF2B [1]. As a result, global protein synthesis is inhibited [2].

Phosphorylation of eIF2α is mediated by different kinases that respond to specific environmental stresses [3]: 1) The general control non-derepressible-2 (GCN2), which is activated during amino acid starvation; 2) the protein kinase activated by double strand RNA (PKR), which plays a key role in the cellular anti-viral response; 3) the PKR-like ER kinase (PERK), which responds to protein misfolding in the endoplasmic reticulum; and 4) the heme-regulated inhibitor (HRI) that limits protein synthesis during heme-deficiency.

The response to stress via phosphorylation of eIF2α is conserved from yeast to mammals and has been shown to underlie cell differentiation in protozoan parasites. For example, phosphorylation of eIF2α by different forms of stresses results in the differentiation of *Toxoplasma gondii,* the agent of toxoplasmosis, into quiescent stages, which are responsible for microbial persistence and latent infection (Narasimhan et al., 2008). Also, phosphorylation of eIF2α of *Plasmodium falciparum* is required in the formation of sporozoytes in the salivary gland of the insect vector [4], [5]. Recently, the importance of eIF2α phosphorylation for the intracellular differentiation of *Leishmania* was demonstrated. In this case, mutant promastigotes that have impaired eIF2α phosphorylation during ER stress, showed delayed differentiation into amastigotes grown axenically, or within macrophages [6].

The developmental transition in *T. cruzi*, a protozoan parasite from the order Kinetoplastida, involves stress responses. A variety of conditions can initiate the process of metacyclogenesis, including changes in pH and osmolarity, presence of mammalian serum [7], haemolymph components [8], gut extracts [9], metabolic stress, and cAMP [10]. Metacyclogenesis can also be reproduced *in vitro* when epimastigotes are transferred to a poor medium containing no proteins, amino acids or sugars (triatomine artificial urine; TAU medium), and then incubated in TAU medium supplemented with amino acids and glucose [11].

Our previous work indicated that trypanosomes encode three potential eIF2 kinases (eIF2K1-K3), and we characterized the *Trypanosoma brucei* TbeIF2K2, showing that this kinase is able to phosphorylate yeast and mammalian eIF2α at Ser^51^. It also phosphorylates the highly unusual form of eIF2α found in trypanosomes specifically at residue Thr^169^ that corresponds to Ser^51^ in other eukaryotes [12]. These data suggested that key features of the eIF2-signaling pathway are conserved in trypanosomes. Because they lack extensive transcriptional control mechanisms, it is thought that regulation at the translational level is critically relevant to the biology of these parasites.

In this study, using specific antibodies that recognize the phosphorylated Thr^169^ residue of *T. cruzi* eIF2α, we show that this phosphorylation mediates translational control in response to nutritional stress. Importantly, we also found that phosphorylation of Tc-eIF2α at Thr^169^ residue controls metacyclogenesis, since a mutation of Thr^169^ to Ala residue impaired differentiation. Interestingly, epimastigotes overexpressing wild type Tc-eIF2α differentiate at higher frequency compared to epimastigotes overexpressing the Tc-eIF2α^Thr169Ala^ mutant. The eIF2α pathway is then proposed to participate in the adaptive response of *T. cruzi* to nutritional stress, contributing to parasite differentiation to disease-causing metacyclic-trypomastigotes.

## Materials and Methods

### Parasite cultures and metacyclogenesis

*T. cruzi* Dm28c and Y epimastigotes were cultured in liver infusion tryptose (LIT) medium at 28°C. Metacyclogenesis was performed as described [11]. In brief, exponentially growing epimastigotes (density of 5×10^7^ parasites ml^− 1^) were collected by centrifugation at 2,000 g for 10 min at room temperature, and were subjected to nutritional stress for 2 h at 28°C in TAU medium (190 mM NaCl, 17 mM KCl, 2 mM MgCl_2_, 2 mM CaCl_2_, 8 mM phosphate buffer pH 6.0) at a density of 5× parasites ml^−1^. The parasites were diluted to 5 × 10^6^ parasites ml^− 1^ in TAU supplemented with 2.5% sodium bicarbonate, 500 U ml^− 1^ penicillin, 10 mM L-proline, 50 mM L-glutamic acid, 2 mM L-aspartic acid, 10 mM glucose and incubated at 28°C for the designated length of time according to the experimental protocol.

### Reagents, synthetic peptides and antibodies

A peptide corresponding to the *T. cruzi* eIF2α motif PYTEIT^P^R, containing the phosphorylated threonine at position 169, and a peptide carrying the corresponding non-phosphorylated threonine were synthesized by Dr. Maria Aparecida Juliano (UNIFESP, São Paulo, Brazil) as described [13]. Antibodies were obtained in rabbits by subcutaneous immunization with the phosphorylated synthetic peptide conjugated to keyhole limpet hemocyanin (Imject Maleimide Activated mcKLH-Pierce) in Freund's complete adjuvant. Sera were obtained after two additional injections of the conjugate emulsified in incomplete Freunds' adjuvant in 3-week intervals between each dose. Monospecific antibodies from the strongest reacting antiserum were purified by chromatography through an iodoacetyl-agarose column (Sulfolink Immobilization Kit for Peptides, Pierce) containing the immobilized phosphopeptide as described by the manufacturer. Specific antibodies were eluted with 100 mM glycine (pH 2.5), neutralized to pH 7.4 and stored at 4°C. The antibodies were further purified by passage through a column containing the non-phosphorylated peptide (PYTEITR) conjugated to BSA. The specificity of purified antibodies, named anti-Tc-eIF2αP, was determined by ELISA and immunoblotting assays. Antibodies that recognize total Tc-eIF2α were obtained in rabbits immunized with a *T. brucei* eIF2α recombinant protein purified from *E. coli* [12]. The monoclonal antibody for *T. cruzi* metacyclic trypomastigote mucins (10D8) was kindly provided by Dr. Nobuko Yoshida (UNIFESP, São Paulo, Brazil) [14].

### Generation of Tc-eIF2α overexpressing parasites

The *T. cruzi* wild-type eIF2α coding sequence was amplified from genomic DNA of DM28c strain using oligonucleotides eIF2aF (5′-CCTGTATTTTCAGGGCATGGCGTCGCATGGCCCAGTGAA-3′) and eIF2aR (5′-GTACAAGAAAGCTGGGTATTTCAATCTGTCTCATCATCATC-3′) based on the CL-Brener sequence with recombination sites appended to the 5′ end for insertion into the pDONR221 vector (Gateway^TM^ Platform). The amplified fragment was inserted into pDONR221 (entry vector) and transferred to pDEST17 according to the manufacturer's protocol (Invitrogen). The insert was removed from by XbaI and HindIII digestion and transferred to the p33 vector [15] linearized with the same enzymes. The introduction of the T^169^A substitution in Tc-eIF2α was performed by overlapping PCR, of two PCR fragments, using the oligonucleotides: Tc-eIF2 FX (5′-TCTAGAATGTCGTACTACCATCACC-3′) and Tc-eIF2 Rbg (5′-GCAACGTGACATACAATGGATCTCACC-3′) for a 788 base pair 5′ fragment, and Tc-eIF2 TA169 (5′-CCGTACACGGAAATTGCGAGGATCCGCATTCG-3′) and Tc-eIF2 RH (5′-AAGCTTTCAATCTGTCTCATCATC-3′) for a 765 base pair 3′ fragment. The products were cloned into the pGEM®-T easy vector systems (Promega) and the constructions were confirmed by sequencing. The sequences encoding the wild type and the mutant Tc-eIF2α were transferred as XbaI-HindIII fragments into the p33 vector [15]. Both constructs encoded the His-tag, derived from the pDEST-17 vector, fused to the N-terminus of eIF2α. These sequences were submitted to GenBank (JN590254 and JN590255).

### Parasite transfection

*T. cruzi* epimastigote cells of Y strain were transfected with the plasmids using a Gene Pulser Electroporator (Bio-Rad Laboratories) under the following conditions: 2.5×10^8^ parasites grown in LIT medium at 28°C were harvested by centrifugation (2,000 g, 2 min), washed with PBS and resuspended in 400 μl of electroporation buffer (137 mM NaCl, 21 mM HEPES, pH 7.0, 5.5 mM Na_2_HPO_4_, 5 mM KCl, 0.77 mM glucose). The cell suspension was mixed with 50 μg of plasmid DNA and electroporated in 0.4 cm gap cuvettes (Bio-Rad Laboratories) with a single discharge of 450 V and 500 μF generating a time constant of about 5 ms. After electroporation, parasites were left to recover for 48 h at 28°C in LIT medium, followed by selection in the same medium containing 500 μg ml^−1^ geneticin G418 (Invitrogen). A p33-GFP (green fluorescent protein) construct was used as a control for transfection efficiency [15].

### Tc-eIF2α phosphorylation detection and protein labeling with [^35^S] Methionine

Phosphorylation of Tc-eIF2α was monitored by Western blotting. *T. cruzi* extracts (10^6^ parasites μl^−1^) were separated by electrophoresis using a 10% polyacrylamide gel in the presence of 0.1% SDS (SDS-PAGE). Proteins were transferred to nitrocellulose membranes, incubated with 5% non-fat milk in TBS (pH 7.8) containing 0.1% Tween-20 (TBS-T) for 2 h and probed with either rabbit anti-Tc-eIF2α antibody (diluted 1:1,000) or anti-phospho-specific (T^169^) Tc-eIF2αP antibody (diluted 1:200), followed by Protein A-peroxidase conjugate (GE Healthcare). Bound antibodies were visualized using the Immobillon Western blotting substrate (Millipore).

For radiolabeling experiments, epimastigote cells (10^9^) were incubated in 5 ml of RPMI, TAU or TAU3AAG supplemented with 200 μCi of Express [^35^S]-methionine (PerkinElmer) for 2 h at 28°C. Then, 5×10^8^ cells were harvested and lysed in 50 μl of SDS-PAGE sample buffer, boiled for 5 min and 10 µl were fractionated on 10% SDS-PAGE. After Coomassie staining, the gels were incubated in Amersham Amplify^TM^ Fluorographic Reagent (GE Healthcare) for 30 min, dried and exposed to Kodak X-ray film at −70°C for detection of [^35^S]-labeled proteins. For the determination of [^35^S]- methionine incorporation into proteins parasites incubated in RPMI, TAU or TAU3AAG in the presence of radiolabelled methionine for different times were washed in PBS, resuspended in 20 mM Tris-HCl (pH 8.0), 150 mM NaCl, 2 mM MgCl_2_ in the presence of a protease inhibitor cocktail (Complete, Roche) and lysed by freezing and thawing. The insoluble fraction obtained from a trichloroacetic acid (TCA) extraction was recovered on a membrane disc filter by vacuum filtration. After rinsing with a 10% TCA solution and then with 95% ethanol the membrane disc filters were air-dried and placed in a scintillation tube for radioactivity count.

### *In vitro* dephosphorylation assays with lambda protein phosphatase

For dephosphorylation assays, a soluble fraction of *T. cruzi* epimastigotes extract was prepared as described [12]. *T. cruzi* epimastigotes were washed in PBS and resuspended in 20 mM Tris-HCl (pH 8.0), 150 mM NaCl, 2 mM MgCl_2_, 2 mM EGTA, 2 mM benzamidine, 1 µg ml^−1^ of leupeptin, 4 µg of aprotinin ml^−1^ and 10 µg ml^−1^ of pepstatin A in the absence of protein phosphatase inhibitor cocktail to an equivalent of 3×10^6^ parasites per microliter. After freezing and thawing, the suspension was centrifuged at 10,000 g for 15 min and the supernatant was kept as soluble material. A portion of the lysate containing 20 µg of protein was incubated with 100 U of lambda protein phosphatase (New England Biolabs) in 30 µL of lambda-PPase buffer (50 mM HEPES, 100 mM NaCl, 2 mM DTT, 0.01% Brij 35 pH 7.5, 2 mM MnCl_2_) at 30°C for 1 h. Negative controls were performed in the same experimental conditions without protein phosphatase, or with heat inactivated protein phosphatase (65°C for 1 h).

### Polysome profile analysis

*T. cruzi* polysomes were isolated and separated on sucrose gradients as described [16], [17]. Cells (1×10^9^) in 20 ml of the indicated medium were incubated with 100 µg ml^−1^ cycloheximide for 5 min at 28°C. They were then kept on ice for additional 10 min, pelleted by centrifugation and washed with cold PBS buffer supplemented with 100 µg ml^−1^ cycloheximide. After centrifugation at 3,000 g for 10 min at 4°C the pellet was resuspended in 500 µl buffer A (10 mM Tris, pH 7.4, 300 mM KCl, 10 mM MgCl_2_ and 1 mM DTT) containing 100 µg ml^−1^ cycloheximide, 1% (v/v) Triton X-100 and 0.2 M sucrose [18]. The suspension was homogenized by repeatedly inverting the tubes and lysis was monitored by phase-contrast microscopy. The lysate was centrifuged at 3,000 g at 4°C, for 2 min. The cleared supernatant was transferred to a new tube and 1 mg ml^−1^ heparin was added. After spectrophotometric quantification, 10 U Abs_260nm_ were layered onto linear 7% to 47% sucrose density gradients prepared in buffer A and centrifuged at 4°C for 2:30 h at 39,000 RPM in a Beckman SW41 rotor. The gradients were collected from the top and the absorbance at 254 nm was monitored in a continuous flow.

## Results

### *T. cruzi* eiF2α is phosphorylated at threonine 169

**Figure 1:**
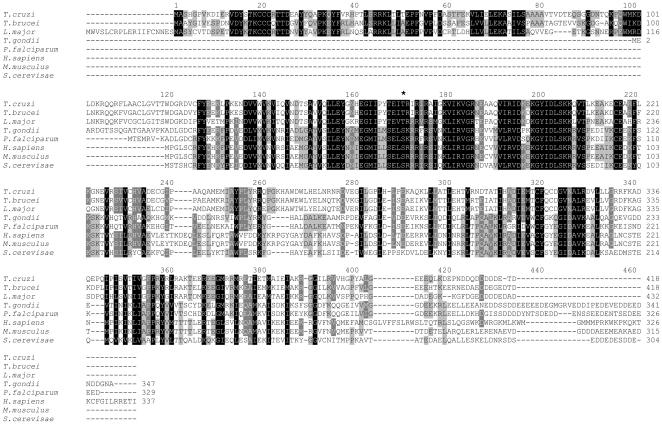
Amino acid sequence alignment of eIF2α orthologues. The sequences shown are from Trypanosomatids (*T. cruzi, T. brucei* and *L. major*), Apicomplexa (*T. gondii* and *P. falciparum*), Human (*H. sapiens*), Mouse (*M. musculus*) and Yeast (*S. cerevisiae*). The black star at the top of the alignment indicates the amino acid that is phosphorylated and that was mutated to alanine in this work. Conserved residues are highlighted.

**Figure 2:**
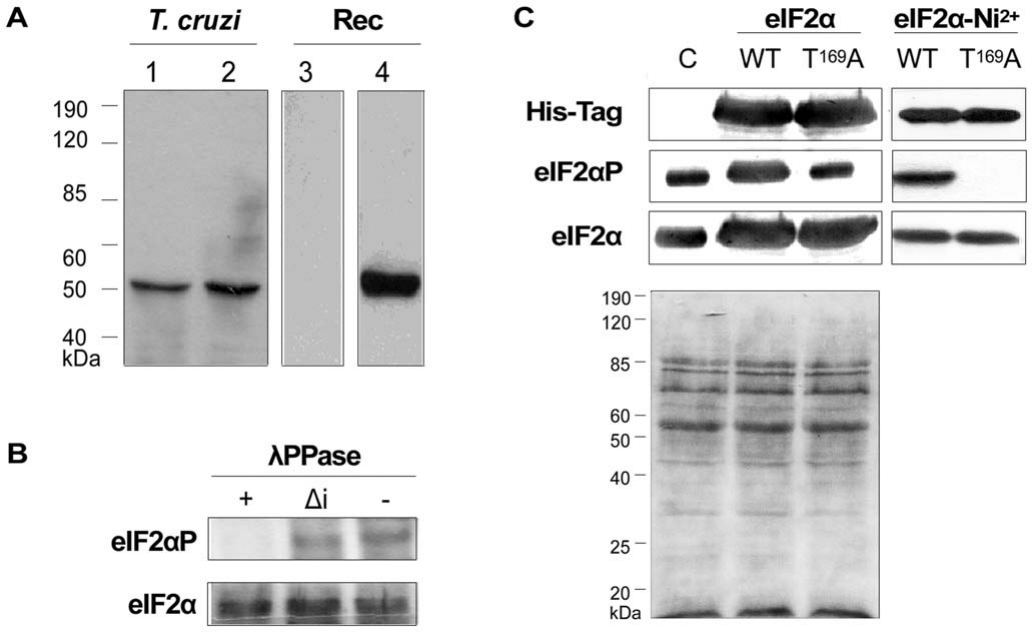
Tc-eIF2α is recognized by antibodies raised against the phosphorylated peptide. (A) Western blot analysis of whole-cell extracts of *T. cruzi* epimastigotes and the purified recombinant His_6_-TceIF2α protein using antibodies raised against the phosphorylated peptide (lanes 1 and 3) and antibodies that recognize total eIF2α (lanes 2 and 4). (B) Soluble extracts of epimastigotes were treated with λPPase (+), heat-inactivated λPPase (Δi) or incubated under the same conditions without phosphatase (−) and then subjected to immunoblotting with anti-phosphospecific, or total Tc-eIF2α antibodies. (C) Equal amounts of control and His_6_-Tagged eIF2α of epimastigotes overexpressing the wild type protein or the Thr^169^Ala mutant protein were subjected to immunoblots using antibodies against the His_6_-Tag, against total eIF2α or against the phosphorylated form of eIF2α. The Ponceau S stained membrane is shown on the bottom panel.

Trypanosomatids encode an ortholog of eIF2α that shares conserved features with other known eIF2α sequences. However, besides an extended N-terminal region with no similarity to any eukaryotic sequence in the NCBI database, eIF2α of trypanosomatids possesses a threonine residue at position 169 (Thr^169^) that corresponds to the serine 51 (Ser^51^) that is phosphorylated in all other eukaryotes (Figure 1A). Comparison of eIF2α sequences from different organisms revealed strong conservation of residues flanking Thr^169^, such as the positively charged residues following the Thr^169^ (Arg^170^, Arg^172^, Arg^174^) and the KGYID_201_ motif, known to play an important role in both translation regulation and Ser^51^ phosphorylation [19]. We have previously shown that Thr^169^ in Tb-eIF2α was specifically phosphorylated by Tb-eIF2K2 *in vitro* [12]. These features indicated that Thr^169^ might be the actual target of stress activated eIF2 kinases that mediate translational inhibition. To study this pathway in trypanosomatids, we then raised antibodies against the *T. cruzi* eIF2α sequence PYTEIT^p169^R in which the T^p169^ represents the phosphorylated threonine. In ELISA assays the affinity-purified antibody showed reactivity to the phosphorylated peptide but not to the unphosphorylated one, or to an unrelated control peptide corresponding to the *T. cruzi* histone H1 [20] in its non- or phosphorylated forms (data not shown). In immunoblots of whole-cell extracts from *T. cruzi* epimastigotes these antibodies recognized a protein with an apparent molecular mass of 50 kDa that migrated at the same position as the protein recognized by the antibodies against total Tc-eIF2α (Figure 2A). The antibodies against TceIF2αT^p169^ did not recognize the recombinant Tc-eIF2α purified from *E. coli* as a His-tagged protein, which is not phosphorylated. To ascertain that the antibodies reacted specifically with the phosphorylated form of eIF2α, *in vitro* dephosphorylation assays were then performed with the soluble fraction of *T. cruzi* epimastigotes cell extract. As shown in Figure 2B treatment with active lambda protein phosphatase (λPPase), but not with the heat-inactivated enzyme, abolished the recognition of the eIF2α protein by these antibodies.

To further confirm the specificity of the antibodies and to assess the role of Tc-eIF2α phosphorylation in *T. cruzi*, we generated parasite lines that, in addition to the endogenous chromosomally encoded eIF2α, overexpressed the wild type and a mutant form of eIF2α in which the Thr^169^ residue was substituted to alanine (Tc-eIF2αT^169^A). Both proteins were expressed as fusions to a His-tag. The expression of the fusion proteins before and after purification with a nickel chelating resin was confirmed by Western blot with anti-His antibodies (Figure 2C, upper panel). The overexpression of wild type and mutant eIF2α proteins in these parasites was ascertained by probing the same membrane with antibodies against total eIF2α (Figure 2C, bottom panel). When the same membrane was probed with the antibodies against Tc-eIF2αT^p169^, a signal was detected in whole-cell extracts of control parasites and in parasites overexpressing Tc-eIF2αT^169^A, corresponding to basal levels of endogenous eIF2α phosphorylation (Figure 2C, left middle panel). On the other hand, in parasites overexpressing the wild type protein, this signal is stronger. When His-tagged eIF2α protein recovered by Ni^2+^-NTA affinity chromatography were analyzed by Western blot, only the wild type form was recognized by the phospho-specific antibody (Figure 2C, right middle panel). We conclude that *T. cruzi* eIF2α is phosphorylated at T^169^ and is specifically recognized by Tc-eIF2αP antibody.

### Nutritional stress impairs translation initiation and increases phosphorylation of eIF2α

**Figure 3:**
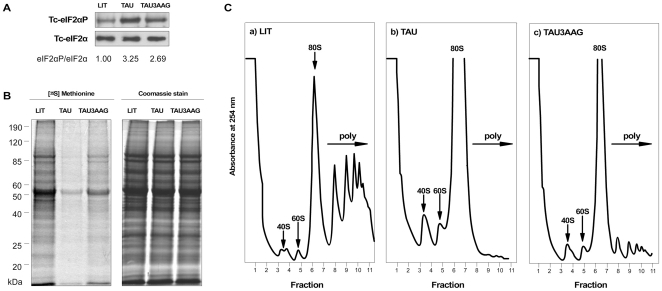
Nutritional stress leads to increased phosphorylation of eIF2α and to inhibition of translation. (A) Immunoblots of equivalent amounts of epimastigotes incubated in rich medium (LIT), or incubated for 2 h in TAU (TAU), or incubated for 2 h in TAU followed by incubation for 2 h in TAU3AAG (TAU3AAG), using antibodies that specifically recognize phosphorylated eIF2α (Tc-eIF2αP) and total Tc-eIF2α. The numbers below each lane show the relative rate of phosphorylated eIF2α and total eIF2α. (B) Incorporation of [^35^S]-methionine by epimastigotes subjected to identical growth conditions as in (A). Samples were resolved on SDS-PAGE for autoradiography (left panel). The Coomassie Blue stained gel is shown on the right panel. (C) Polysome profiles of cell extracts prepared from epimastigotes subjected to identical growth conditions as in (A). A total of 10 A_260nm_ units were fractionated through a 7–47% linear sucrose gradient. The arrows indicate the peaks for the 40S and 60S ribosomal subunits, the 80S monosome peak; the polysomes are indicated with a bracket (poly). This result is an example of similar results obtained in three independent experiments.

Having shown that the antibodies recognized specifically the phosphorylated form of *T. cruzi* eIF2α, we then employed them to assess the phosphorylation of eIF2α in parasites submitted to nutritional stress. Exponentially growing wild type epimastigotes were submitted to nutritional deprivation for 2 h in TAU medium. This experimental condition allows a metabolic synchronization of the cells in such a way that further incubation in a relatively poor medium (TAU3AG - TAU supplemented with glucose, proline, aspartate and glutamate) allows the transformation of epimastigotes into infective metacyclic trypomastigotes within 72 h [11]. Western blot analysis of extracts of exponential epimastigotes incubated in TAU showed a large increase in the levels of phosphorylated Tc-eIF2α relative to parasites maintained in rich medium (LIT) (Figure 3A). Total amounts of Tc-eIF2α were similar in all populations as determined with the antibodies that recognize total eIF2α (Figure 3A). When epimastigotes subjected to nutritional stress were allowed to recover for 2 h in TAU3AAG medium, a reduction in the levels of Tc-eIF2α phosphorylation was observed (Figure 3A). Identical results were obtained in three independent experiments and with different transfectants revealing an increased ratio of phosphorylated over total eIF2α. These experiments validate that nutritional deprivation elicits eIF2α phosphorylation in *T. cruzi* and that addition of the three amino acids and glucose allows for a fast partial dephosphorylation of eIF2α.

To study the effects of these nutritional stress conditions on protein synthesis, we initially investigated the incorporation of radiolabelled amino acids into newly synthesized proteins. Control and experimental parasites were incubated with [^35^S] methionine during the 2 h period of stress treatment (or mock treatment). The autoradiography of SDS-PAGE showed that the overall protein synthesis was greatly reduced in parasites maintained in TAU medium compared to parasites maintained in LIT medium (Figure 3B). When TAU-stressed epimastigotes were then incubated for an additional 2 h in TAU3AAG, incorporation was increased. The kinetics of [^35^S]-methionine incorporation during growth and induction of metacyclogenesis confirmed this data (Figure S1). Epimastigotes kept for 2h in TAU medium showed a smaller incorporation than parasites cultivated in LIT or maintained in TAU3AAG for up to 72 h.

To directly address whether these nutritional conditions affected the initiation step of translation, as would be expected from increased Tc-eIF2α phosphorylation, we studied the polysome profiles of exponential epimastigotes subjected to incubation in TAU and TAU3AAG media, and control parasites grown in LIT. This technique separates ribosomal subunits, ribosome monosomes and polysomes (*i.e.* polyribosomes) according to their densities on a sucrose gradient. Efficient translation results in mRNAs bound to many ribosomes, called polysomes, whereas poor translation initiation causes a decrease in size and abundance of polysomes and a concomitant increase in 80S ribosomes (monosomes). We observed that incubation of exponential epimastigotes for 2 h in TAU medium completely abolished the polysomal fractions and enhanced the amount of monosomes, indicating a very strong inhibition of translation initiation (Figure 3C), as also found by others [21]. This observation is in line with the reduction of global protein synthesis determined by metabolic labeling with [^35^S]-methionine and with the increased phosphorylation of eIF2α observed in parasites incubated in TAU. Upon transfer to TAU3AAG medium, required for efficient metacyclogenesis, we observed a significant recovery of translation with the reappearance of polysomes (Figure 3C). Again, this reflects the increase in [^35^S]- methionine incorporation and the decrease in the relative levels of eIF2α phosphorylation observed in the same experimental conditions. These results then correlate decreased protein synthesis observed upon nutrient deprivation with poor translation initiation that result from the phosphorylation of eIF2α.

### Tc-eIF2αT169A maintains efficient translation during nutritional stress

**Figure 4:**
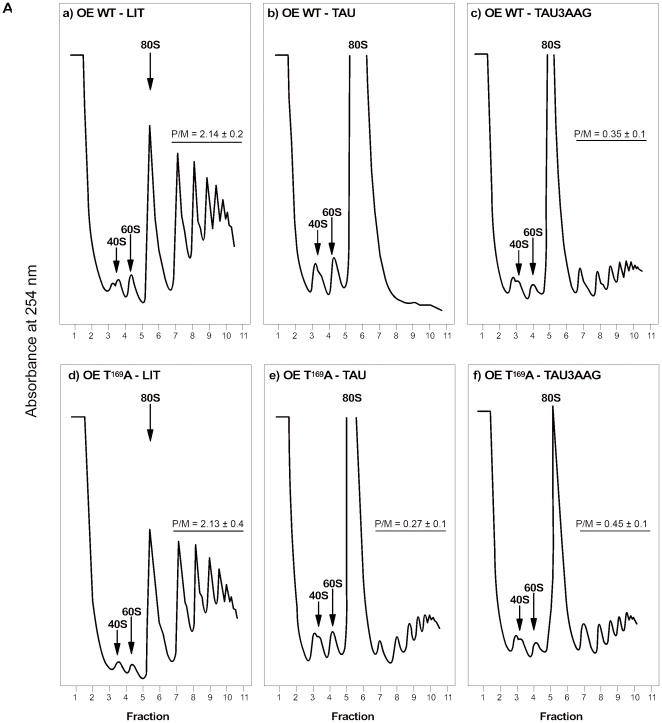
Overexpression of Tc-eIF2αT^169^A protects cells from inhibition of translation initiation during nutritional stress. (A) Polysome profiles of parasites overexpressing the wild type eIF2α (OE WT; panels a–c) and mutant Tc-eIF2αT^169^A (OE T^169^A; panels d-f) grown in LIT medium (panels a and d) or maintained for 2 h in TAU (panels b and e) or for additional 2 h in TAU3AAG medium (panels c and f). The polysome/monosome ratio was determined by measuring the area of the polysome peaks and of the 80S peak using the ImageJ software (developed and maintained by the National Institutes of Health, Bethesda, MD). For OE T^169^A - TAU (panel e) the monosome/polysome ratio was obtained by extrapolation of the 80S area. Shown are representative profiles from three independent experiments.

To directly address whether phosphorylation of eIF2α was responsible for the impairment in translation initiation observed in parasites incubated in TAU, we analyzed the polysome profiles of parasites overexpressing the mutant Tc-eIF2αT^169^A (OE-T^169^A) and as a control, the parasites overexpressing the wild type protein (OE-WT). Parasites were incubated in LIT, or subjected to nutritional stress in TAU or in TAU followed by TAU3AAG media. The profile of the OE-T^169^A parasites grown in LIT was similar to that of the OE-WT kept under the same experimental condition (Figure 4, panels a and d). Importantly, while OE-WT epimastigotes maintained for 2 h in TAU revealed a complete disappearance of the polysomes accompanied by a large increase in the amount of free 80S subunits, the OE-T^169^A parasites maintained a significant level of translation, as evidenced by the presence of polysomes (Figure 4, panels b and e). Nevertheless, the parasites overexpressing the mutant Tc-eIF2αT^169^A are not fully competent for translation at starvation possibly due to the residual amount of endogenous phosphorylated eIF2α, as demonstrated by the monosomes/polysomes ratios. Alternatively, it is possible that additional mechanisms that impair translation may be at play under this strong stress situation. A shift to TAU3AAG for 2 h resulted in a decrease in 80S monosome peak and increased polysome levels in the OE-WT parasites, (Figure 4, panel c), while a more pronounced decrease of 80S peak was observed for OE-T^169^A (Figure 4, panel f). These polysome profile data support the conclusion that phosphorylation of the Threonine-169 residue of Tc-eIF2α that occurs upon nutritional stress is a primary mechanism by which translation inhibition occurs in these parasites.

### Tc-eIF2α phosphorylation regulates metacyclogenesis

**Figure 5:**
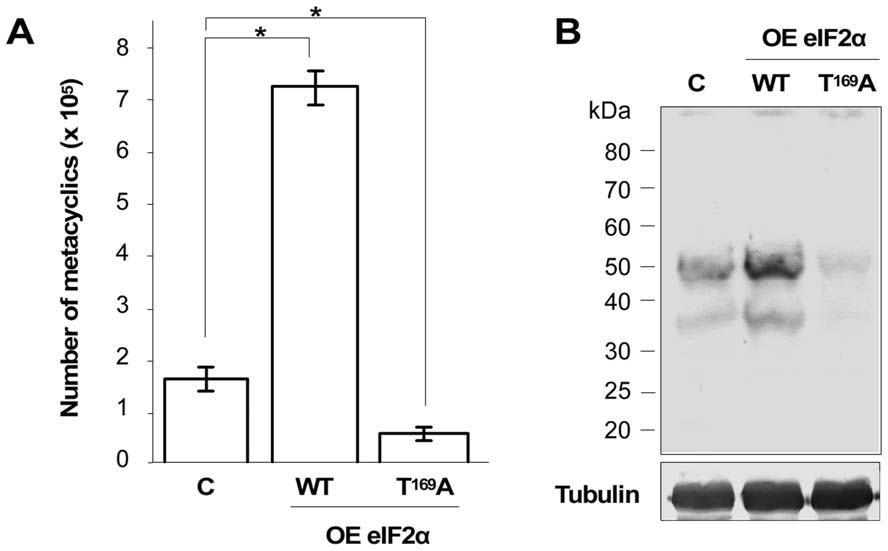
Phosphorylation of eIF2α is essential for the differentiation of epimastigotes into metacyclic trypomastigotes. (A) Number of metacyclic trypomastigotes on the supernatant of control wild type parasites (c), of parasites overexpressing the wild type eIF2α protein (OE WT) and of parasites overexpressing the mutant eIF2αT^169^A protein (OE Tc-eIF2αT^169^A) subjected to the differentiation protocol of incubation in TAU medium for 2 h followed by 72 h of incubation in TAU3AAG medium. The bars represent the mean ± SD from triplicates. *T-student*, p<0,01 (n = 3) (B) Immunoblot analysis of whole cell extracts from parasites recovered from the supernatant of differentiating cultures using the monoclonal antibody 10D8 (top panel) and anti-tubulin antibodies for normalization (bottom panel).

Our findings that phosphorylation of Tc-eIF2α is increased in TAU prompted us to examine if this event is required to trigger the differentiation of epimastigotes into metacyclic trypomastigotes *in vitro* (metacyclogenesis). Parasites overexpressing Tc-eIF2α or Tc-eIF2αT^169^A grown in LIT medium were stressed for 2 h at 28°C in TAU and then submitted to differentiation in TAU3AAG medium for 72 h. Under this condition, the parasites attach to the substrate and metacyclics are progressively released [11]. After this period, the yield of metacyclogenesis was determined by counting the number of metacyclic trypomastigotes on the supernatant of cultures. As control for metacyclogenesis, we employed non-transfected parasites subjected to identical conditions. Surprisingly, parasites that overexpress the wild type eIF2α showed an enhanced yield of metacyclics when compared to the non-transfected parasites (≈4.5 fold-higher) (Figure 5A). Significantly, moreover, the parasites overexpressing the mutant protein showed diminished amount of metacyclics, when compared to the parasites overexpressing the wild type protein, and even when compared with the non-transfected line. To ascertain the differentiated phenotype of the parasites, we performed Western blot analysis of total cells collected from the supernatants after 72 h of differentiation using the monoclonal antibody 10D8 that recognizes the 35/50 kDa glycoconjugate specific of metacyclic forms [14]. The result shows that polypeptides of 35- and 50 kDa have their expression increased in wild-type Tc-eIF2α parasites and are almost absent in Tc-eIF2αT^169^A mutants (Figure 5B). Consistent with eIF2α phosphorylation levels and with the polysomes profiles, a deficit in this stress response corresponded to a deficit in metacyclogenesis. These data taken together clearly show that differentiation of *T. cruzi* into its infective form requires the inhibition of translation by phosphorylation of eIF2α, triggered by nutritional deprivation.

## Discussion

This study demonstrates for the first time that the eIF2α stress-response pathway is required for the *in vitr*o differentiation of *T. cruzi* into its infective form. In addition, we have unambiguously demonstrated that in this organism eIF2α is phosphorylated *in vitro* at the Thr^169^ residue during nutritional deprivation. This residue corresponds to the invariable phosphorylated serine residue in eIF2α found in all other eukaryotes analyzed to date. Trypanosomatid's eIF2α contains conserved motifs near this residue that further support that Thr^169^ is the target of phosphorylation. Residues Lys^197^-Asp^201^ in Tc-eIF2α correspond to residues Lys^79^ through Asp^83^ (sequence KGYID) involved in both kinase and eIF2B recognition in typical eIF2α [22], [23]. Interestingly, the highly conserved LSRRR motif that encompasses the phosphorylated Ser^51^ residue in all other eukaryotes is also found at residues 42 to 46 in the N-terminal extension of *Trypanosoma* eIF2α (Figure 1B). A secondary structure prediction of this N-terminal extension (data not shown) indicated that it could adopt the S1 fold that provides the fitting of the eIF2α phosphorylation site loop to the kinase catalytic center [24]. However, because of the lack of the KGYID-like motif near this region, it is highly unlikely that this serine residue may be phosphorylated by the typical eIF2α kinases. The relevance of these conserved residues in the N-terminal extension of trypanosomatids eIF2α is not known.

Our data clearly showed that phosphorylation of Tc-eIF2α at Thr^169^ results in inhibition of translation initiation in this organism. Furthermore, by overexpressing a mutant Tc-eIF2α that is unable to be phosphorylated by the typical eIF2 kinases, we have provided evidence that this modification is required for metacyclogenesis.

In the blood-sucking Triatomine insects many different developmental stages of *T. cruzi* appear, and replication and differentiation occur in distinct compartments along the digestive tube [25]. The successful development of *T. cruzi* infection in the gut of the insect vector is dependent on a range of biochemical and physiological factors. Usually, the importance of the nutritional state is emphasized in the invertebrate host: the lack of nutrients in the intestinal tract affects not only the population of *T. cruzi*, but also the different developmental stages [25]. The exact molecular mechanism controlling the stress-induced metacyclogenesis is still obscure. Nevertheless, it is reasonable that nutrient availability decreases, as the parasite is ready to be released. Ability to respond to nutritional stress by phosphorylation of eIF2 renders *T. cruzi* able to undergo this differentiation process, critical for parasite infectivity. Our data thus shed light on a molecular event that is directly involved in promoting metacyclogenesis. In our studies, differentiation from epimastigotes to metacyclic trypomastigotes, induced by nutritional stress, was greatly enhanced by the overexpression of the wild type form of eIF2α, and inhibited by the overexpression of eIF2α containing a single amino acid change. These observations can be interpreted by the drastic increase in the amount of phosphorylated eIF2α upon stress. The phosphorylated form of eIF2-GDP sequesters the limited quantities of eIF2B in a tight complex, preventing the recycling reaction and, consequently, reducing the amounts of eIF2-GTP available for new rounds of initiation. On the other hand, the overexpression of the mutant protein would result in a large availability of eIF2-GTP even under stress conditions. These effects are reflected in the polysome profiles of cells subjected to nutritional stress. The striking effect of a single amino acid change in the response of *T. cruzi* to the *in vitro* differentiation protocol thus highlights the role of eIF2 in metacyclogenesis.

Phosphorylation of the eIF2α can differentially affect translation of mRNAs genome-wide. Certain transcripts may be preferentially translated under conditions that lower the availability of the ternary complex. This is the case of GCN4 in yeast and ATF4 in mammals [2]. These messages are translated by a non-conventional mechanism that involves the presence of small open reading frames (uORF) in the leader sequence of their mRNA's, which impedes the translation the ORF encoding the main protein. Under conditions that increase phosphorylation of eIF2α, the resulting lower availability of the ternary complex allows the ribosomes to bypass the uORFs and translate the main ORF. It is possible that proteins involved in the parasite differentiation may be preferentially synthesized when eIF2α phosphorylation is increased. To confirm this hypothesis evaluation of translation of individual mRNAs during nutritional stress and mock treatment would be effective to understand the underlying mechanisms by which Tc-eIF2α phosphorylation recalibrates the parasite gene expression network towards the differentiation program.

It should be noticed that the parasites used for the overexpression experiments still encoded the chromosomal wild type version of eIF2α. This could account for some of the remaining effects of the nutritional stress, both regarding the degree of translation inhibition and the low levels of differentiation observed with the overexpression of the mutant eIF2α. However, we cannot rule out multiple, redundant and overlapping mechanisms, besides eIF2α phosphorylation, that may also function to promote the differentiation process. Indeed, the protein synthetic capacity of eukaryotic cells to respond to physiological stimuli can also be regulated by other mechanisms. For example, nutrient availability is known to affect the loading of the ribosomes onto the mRNAs, in a mechanism mediated by the kinase TOR, orthologues of which have been described in the trypanosomatids.

Changes in gene expression during stress conditions are universal phenomena that occur to favor cellular survival. In trypanosomes, gene expression is controlled post-transcriptionally and at the level of translation initiation [26], [27]. Moreover, in *T. cruzi* and *T. brucei*, relocation of polysomal mRNA into large cytoplasmic stress granules has been observed when parasites are submitted to metabolic stress [16], [28] and heat shock insult [29], respectively. Analysis of the protein composition of trypanosomal mRNA granules indicates that they contain orthologous proteins to those present in P-bodies and stress granules (SGs) from metazoan organisms [28]. These granules contain stored mRNAs and share components that are involved in the initiation of translation, including eIF2α [16], [29]. Indeed, in mammalian cells, the formation of stress granules is dependent on the phosphorylation of eIF2α at the Ser^51^. It would be interesting to determine whether eIF2α phosphorylated at Thr^169^ is relocated onto stress granules in *T. cruzi* upon TAU incubation, although this does not seem to be the case for *T. brucei*, after heat shock [29].

Cell differentiation in response to stress via phosphorylation of eIF2α has been described in others parasites. In *Leishmania infantum* phosphorylation of eIF2α plays an important role in the differentiation of promastigotes to amastigotes without cells and also at early stages following macrophage infection [6]. When *Toxoplasma gondii* tachyzoites (the rapid replicative forms of this parasite) are subjected *in vitro* to heat shock, alkaline pH, or other forms of stress, they phosphorylate eIF2α and start expressing cyst wall antigens of bradyzoites, the parasite's latent forms [4], [30]. Transformation from gland latent sporozoite into liver stages in *Plasmodium berghei* is also dependent on the phosphorylation status of eIF2α, involving this signaling cascade in parasite's survival and recrudescence of the disease [5]. While in *T. gondii*, two eIF2α kinases were characterized (TgIF2K-A and TgIF2K-B localized to the ER and cytosol, respectively) and described as involved in the phosphorylation of Tg-eIF2α, in *P. berghei* three eIF2 kinases were described (IK1, IK2 and PK4) [5], [31], [32]. Previous work reported the presence of three putative eIF2α kinases in *T. brucei* (TbeIF2K1-K3) being one of them, TbeIF2K1, a possible GCN2 orthologue [12]. *Leishmania* encodes a homologue of the ER stress inducible PERK eIF2α kinase but while in *T. brucei* eIF2K2-PERK kinase is localized in the flagellar pocket [12] in *Leishmania spp*. it is associated with the ER [6]. Counterparts of the three eIF2 kinases described in *T. brucei* are also present in the genomes of *T. cruzi* and bioinformatics search revealed two potential homologues of TbeIF2K3 in this parasite [33]. Collectively, these data suggest that eIF2α kinases are not only present but also active in *T. cruzi*. The eIF2α kinase, or kinases, activated under the conditions used here has yet to be determined. The GCN2 ortholog might be a likely candidate. Whether this signaling mechanism influences other *T. cruzi* differentiation events, i.e., the transformation of infective stages to intracellular amastigotes after cell invasion, probably driven by acidification [34], or the differentiation of intracellular forms in newly infective forms remains to be investigated. The present results opens therefore, new tools and possibilities to understand these processes, which can be relevant in establishing new targets for chemotherapy, an important need to combat Chagas' disease.

## Supporting Information

Figure S1 **Nutritional stress leads to translation repression in epimastigotes.** Kinetic of [^35^S]-methionine incorporation in growing epimastigotes (LIT, 0–72 h), epimastigotes maintained for 2 h in TAU medium (TAU, 2 h) and epimastigotes incubated in the differentiating TAU3AAG medium (TAU3AAG, 24–96 h). These data were obtained in one experiment performed in triplicate.
(TIF)
