# Supplementary figures and images for: Protein Synthesis Attenuation by Phosphorylation of eIF2α Is Required for the Differentiation of Trypanosoma cruzi into Infective Forms
Source: PLoS One. 2011 Nov 16;6(11):e27904. doi: 10.1371/journal.pone.0027904 (PMC3218062; doi:10.1371/journal.pone.0027904)

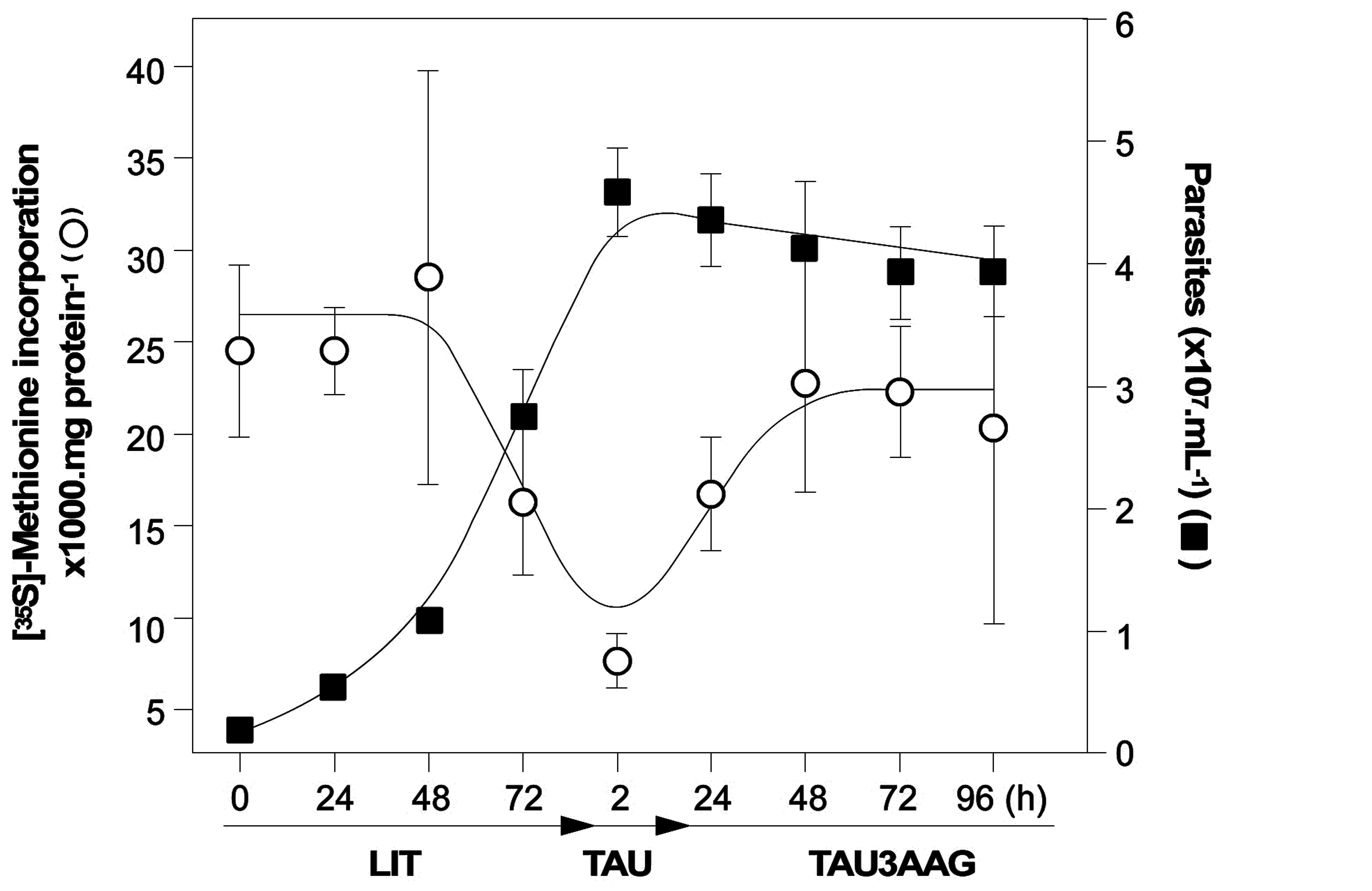

Supplement: Figure S1 — Nutritional stress leads to translation repression in epimastigotes. Kinetic of [35S]-methionine incorporation in growing epimastigotes (LIT, 0–72 h), epimastigotes maintained for 2 h in TAU medium (TAU, 2 h) and epimastigotes incubated in the differentiating TAU3AAG medium (TAU3AAG, 24–96 h). These data were obtained in one experiment performed in triplicate. (TIF) [file pone.0027904.s001.tif]
